# Independent and interactive contributions of cerebrovascular disease, neuroinflammation, and tau pathophysiology to Alzheimer’s disease‐related diagnostic conversion in adults with Down syndrome

**DOI:** 10.1002/alz.087570

**Published:** 2025-01-09

**Authors:** Natalie C. Edwards, Patrick J. Lao, Mohamad J. Alshikho, Batool Rizvi, Lisi Flores Aguilar, Melissa Petersen, Sid E. O'Bryant, Dana Tudorascu, Benjamin L Handen, Jose Gutierrez, Donna M. Wilcock, Elizabeth Head, Adam M. Brickman

**Affiliations:** ^1^ Columbia University Irving Medical Center, New York, NY USA; ^2^ Taub Institute for Research on Alzheimer’s Disease and the Aging Brain, Vagelos College of Physicians and Surgeons, Columbia University, New York, NY USA; ^3^ Columbia University, New York, NY USA; ^4^ Taub Institute for Research on Alzheimer's Disease and the Aging Brain, New York, NY USA; ^5^ University of California, Irvine, Irvine, CA USA; ^6^ University of North Texas Health Science Center, Fort Worth, TX USA; ^7^ University of Pittsburgh, Pittsburgh, PA USA; ^8^ Indiana University School of Medicine, Stark Neurosciences Research Institute, Department of Neurology, Indianapolis, IN USA; ^9^ The UC Irvine Institute for Memory Impairments and Neurological Disorders (UCI MIND), Irvine, CA USA

## Abstract

**Background:**

By age 40 years, adults with Down syndrome (DS) develop Alzheimer’s disease (AD) pathology and progress to dementia in their 60s. Despite minimal systemic vascular risk factors, individuals with DS have MRI evidence of cerebrovascular injury that progresses with AD severity, suggesting an intrinsic vascular component to DS‐AD that may interact with neuroinflammatory processes to promote tau pathology and cognitive decline. In the current study we examined whether cerebrovascular disease (CVD) burden and inflammation/astrocytosis independently and interactively were associated with incident diagnosis among adults with DS.

**Method:**

This study included 149 participants from the Alzheimer Biomarkers Consortium – Down Syndrome (baseline mean age[SD]=44.6[9] years) with available baseline MRI, plasma biomarker data, and at least two time‐points of clinical consensus diagnosis data (i.e., cognitively stable, mild cognitive impairment [MCI], and clinical AD) who were classified as cognitively stable or MCI at baseline. Logistic regression models assessed if baseline small vessel CVD, operationalized as white matter hyperintensity (WMH) volume, and plasma glial fibrillary acidic protein (GFAP), Aβ42/Aβ40, p‐tau217, and neurofilament light (NfL) concentrations are associated with conversion from a milder diagnosis to a more severe clinical diagnosis. Mediation models examined relationships between biomarkers and diagnostic conversion. All models adjusted for study site, sex/gender, latency between visit dates, and age group (below or above/equal to the median age of the sample).

**Result:**

Diagnostic conversion occurred in 26% of the sample. Higher baseline WMH volume (OR 1.08 [1.01, 1.81]), GFAP (OR 1.006 [1.003, 1.01]), and p‐tau217 (OR 20.56 [5.01, 112.43]), but not NfL nor Aβ42/Aβ40 concentrations were associated with higher odds of conversion to more severe cognitive impairment. GFAP concentration mediated the relationship between WMH and diagnostic conversion (ACME 0.05 [0.01, 0.1], p=0.006). P‐tau217 concentration mediated the relationship between GFAP and diagnostic conversion (ACME 0.13 [0.05, 0.23], p=0.004).

**Conclusion:**

Our findings suggest that among individuals with DS, CVD promotes AD‐related clinical progression by increasing astrocytosis which, in turn, promotes tau pathophysiology and downstream MCI and AD incidence. The results implicate CVD and its interface with inflammation as a core feature of AD in DS.

